# An improvement in perception of self-generated tactile stimuli following theta-burst stimulation of primary motor cortex

**DOI:** 10.1016/j.neuropsychologia.2007.04.008

**Published:** 2007

**Authors:** Martin Voss, Paul M. Bays, John C. Rothwell, Daniel M. Wolpert

**Affiliations:** a Sobell Department of Motor Neuroscience, Institute of Neurology, University College London Queen Square, London WC1N 3BG, United Kingdom; b Department of Psychiatry, Charité University Hospital/St. Hedwig Hospital, Turmstr. 21, D-10559 Berlin, Germany; c Institute of Cognitive Neuroscience, University College London 17 Queen Square, London WC1N 3AR, United Kingdom; d Department of Engineering, University of Cambridge, Trumpington Street, Cambridge CB2 1PZ, United Kingdom

**Keywords:** Attenuation of sensory input, Efference copy, Motor cortex excitability, Sensory suppression, Transcranial magnetic stimulation (TMS)

## Abstract

Recent studies have shown that self-generated tactile sensations are perceived as weaker than the same sensations externally generated. This has been linked to a central comparator mechanism that uses efference copy to attenuate the predictable component of sensory inputs arising from one's own actions in order to enhance the salience of external stimuli. To provide a quantitative measure of this attenuation, a force-matching task was developed in which subjects experience a force applied to their finger and are then required to match the perceived force by actively pushing on the finger using their other hand. The attenuation of predictable sensory input results in subjects producing a larger active force than was experienced passively. Here, we have examined the effects of a novel rTMS protocol, theta-burst stimulation (TBS), over primary motor cortex on this attenuation. TBS can alter the excitability of motor cortex to incoming activity.

We show that application of a 20 s continuous train of TBS, that depresses motor cortex, significantly improves performance in a force-matching task. This suggests that the TBS intervention disturbed the predictive process that uses efference copy signals to attenuate predictable sensory input. A possible explanation for the effect is that TBS has a differential effect on the populations of neurones that generate motor output in M1 than on those neural structures that are involved in generating an efference copy of the motor command.

## 1 Introduction

It has been proposed that predicting the consequences of one's own actions is a key feature in sensorimotor control. By using an ‘efference copy’ of the motor command (Holst & Mittelstaedt, 1950) together with an internal model of the environment (Wolpert, Ghahramani, & Jordan, 1995; Wolpert & Miall, 1996) a prediction of the consequences of one's actions can be generated. Such prediction can be used to maintain perceptual stability, control actions in the presence of feedback delays or perform mental practice (for a review see Cullen, 2004).

The prediction of the sensory consequences can also be compared to the actual sensations arising during movement. This comparison can be used to distinguish between sensations that are self-generated and those arising from external sources. Moreover, removing the predicted sensory feedback from the actual feedback provides a signal that reflects unexpected changes in the world and may enhance the salience of externally generated sensations. Consistent with such a mechanism, a number of studies have shown that self-generated tactile sensations are perceived as weaker than the same sensations externally generated (Bays, Wolpert, & Flanagan, 2005; Bays, Flanagan, & Wolpert, 2006; Blakemore, Wolpert, & Frith, 1998; Weiskrantz, Elliott, & Darlington, 1971).

A recent study has investigated sensory attenuation in the perception of self-generated forces (Shergill, Bays, Frith, Wolpert, 2003). A torque motor generated a small constant force on a subject's left index finger, and the subject was then required to reproduce the force they had experienced. When subjects used their right index finger to push on the left index finger to reproduce the experienced force, they produced substantially more force than they had passively experienced. This is consistent with attenuation of the experienced force during direct action of one part of the body on another. In contrast, when they used a joystick in the right hand to control the torque motor to reproduce the experienced force, a novel mapping between actions and sensation in which prediction is unlikely to occur, they could faithfully reproduce the force. Although such sensory attenuation has been investigated psychophysically and shown to be time-locked to the expected time of contact between body parts (Bays et al., 2005), little is known about the neural mechanisms that underlie such a comparison between predicted and actual sensory feedback.

The aim of the present study is to assess the involvement of primary motor cortex (M1) in this mechanism by measuring tactile attenuation before and after administration of a novel transcranial magnetic stimulation (TMS) protocol, theta-burst stimulation (TBS), over MI. Theta-burst stimulation over primary motor cortex has recently been shown to produce long lasting effects on the excitability of the motor cortex as reflected in an increase or decrease in the amplitude of motor evoked potentials (MEP) after TBS (Huang, Edwards, Rounis, Bhatia, & Rothwell, 2005).

If the efference copy signal is a copy of the motor output generated by those population of neurones within MI that can be affected by TBS, then changes to the excitability of MI should affect both the efference copy and the actual motor command equally, and no discrepancy will result. We would therefore expect sensory attenuation to be unaffected.

If efference copy signals do not arise as a copy of the motor output generated from those corticospinal neurones in MI, then an increase or decrease in the excitability of MI will produce a mismatch between the efference copy and the actual motor command sent to the muscles. This will create a discrepancy between the predicted sensory input, based on efference copy, and the actual sensory input related to the motor activity. This kind of discrepancy is usually found when a body part is moved passively by an external force, so predictive processes that normally apply during self-action are likely to be disrupted. For instance, predictive modulation of grip-force is impaired when this kind of sensory discrepancy is introduced, either by amplifying or reducing force feedback (Blakemore, Goodbody, & Wolpert, 1998). Likewise, the central cancellation of self-administered tactile stimuli was reduced when a mismatch between predicted and actual sensory consequences was created by introducing spatial or temporal delays between action and effect (Blakemore, Frith, & Wolpert, 1999).

In the present study, a sensory mismatch caused by an increase or decrease in MI excitability may reduce the normal attenuation of self-generated sensation and hence improve performance on the force-matching task.

## 2 Methods

### 2.1 Subjects

Sixteen healthy right-handed subjects (nine males, seven females; 20–31 years) participated in the experiments. They were naive to the specific purpose of the experiments and gave written informed consent. The study was conducted in accordance with the Declaration of Helsinki and the methodology had been approved by the local ethics committee.

### 2.2 Experimental protocol

**Fig. 1:**
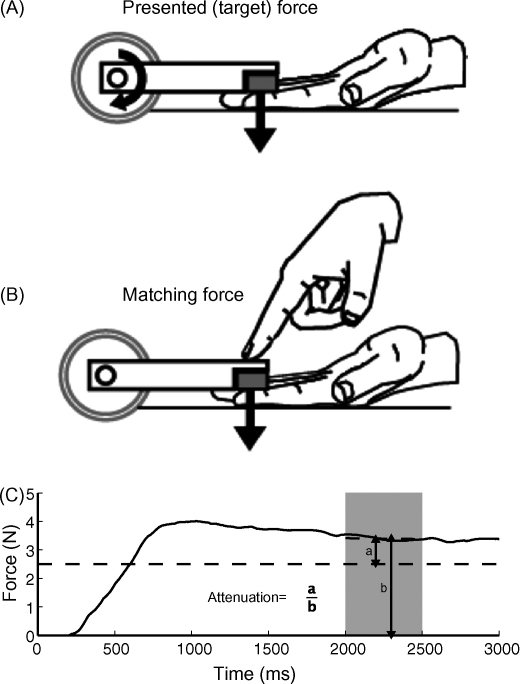
Illustration of the force-matching paradigm. On each trial the torque motor generated a target force between 1 and 3 N on the either the left or the right index finger for 3 s (A). Subjects were then required to reproduce the force by pushing with their opposite index finger (matching force, B). Each subject used both left and right finger to match the target force (blockwise, in a counterbalanced pseudorandom order). The applied forces were measured using a force transducer mounted in the lever of the torque motor. (C) Sample trace of forces generated by one subject (solid line) during a representative trial with a target force generated by the torque motor of 2.5 N (dashed line). The grey area indicates the interval over which the mean subject-generated force was calculated. The “attenuation index” was then calculated as the percentage of the matching force level by which it exceeded the target force level (a/b).

We used a recently developed force-matching task that allows us to quantify the sensory attenuation of self-produced stimuli (Shergill et al., 2003). Subjects rested either their left or right index finger in a moulded support. A force sensor (Nano-17 6-axis F/T sensor, ATI Inc.) rested on the tip of the finger at the end of a lever attached to a torque motor (Fig. 1). To start each trial the torque motor applied one of five constant target forces in the range 1–3 N to the tip of the subject's index finger for 3 s. Following an auditory go-signal, subjects were then required to reproduce the force they had just experienced by pressing with their opposite index finger on the resting index finger through the force sensor. After 3 s an auditory stop-signal indicated the end of the trial.

Subjects completed a total of four blocks of 20 trials each. Throughout each block, they matched the target force by either pushing with their right finger on the resting left finger or vice versa. The order of the first two blocks was randomized. After the second block, subjects received either a 20 s train of *continuous* TBS (eight subjects) or a 190 second train of *intermittent* TBS (eight different subjects) over the left primary motor cortex (see details below). After TBS, subjects sat with their hands completely relaxed for 10 min. Subject then completed the final two blocks which were in the same order as the first two blocks. Each block took 7.5 min to complete.

The matching force level generated by the subject was calculated for each trial by taking the mean force recorded by the force sensor between 2000 and 2500 ms after the go-signal. The attenuation on each trial was then calculated as the percentage of the matching force level by which it exceeded the target force level (see Fig. 1C). This reflects the percentage of the self-generated force that is not perceived. This measure was averaged across trials to give the mean attenuation for each subject and condition.

To investigate the effects of *continuous* and *intermittent* TBS on the amount of attenuation two separate 2 × 2 repeated measures ANOVAs were performed with the factors “time” (pre/post-TBS) and “finger” (left/right finger active).

### 2.3 TMS procedure

Focal transcranial magnetic stimulation (TMS) with a figure-of-eight-coil (Magstim Comp., Dyfed, UK; diameter of each coil was 7 cm) was used to elicit magnetic evoked potentials in the first dorsal interosseous muscle of the relaxed right hand. The coil was kept tangential to the head. Orientation was approximately perpendicular to the central sulcus of the dominant left cortex (45° from the anterior–posterior axis) with the handle pointing posteriorly. The motor evoked potentials were recorded with Ag/AgCl sintered electrodes placed over the belly and tendon for the hand muscles (time constant: 3 Hz, low-pass filter: 3kHz, sampling rate: 10 kHz). First, the optimal spot on the skull was determined as the cortical site where muscle responses could be evoked with the lowest stimulator output intensity. This position was marked with ink to allow an exact re-positioning of the coil throughout the experiments. After finding the optimal spot, subjects were asked to produce a tonic pinch grip between the index finger and thumb. The active motor threshold was defined as the minimum single pulse intensity required to produce an MEP of greater than 200 mV on more than five out of 10 trials from the contralateral FDI while the subject was maintaining a voluntary contraction of about 20% of maximum using visual feedback.

During the TMS-intervention, cortex stimulation was carried out using a recently developed stimulation protocol in which trains of TMS were delivered in bursts of three pulses at 50 Hz (“Theta-Burst Stimulation”, TBS (Huang & Rothwell, 2004; Huang et al., 2005). The bursts were either delivered continuously at a rate of 5 Hz (200 ms gap between each burst) at 80% active motor threshold over a 20 s period (*continuous TBS*) or in a 2 s train of TBS repeated every 10 s for a total of 190 s (600 pulses, *intermittent TBS*). This stimulation protocol is known to either depress (*continuous TBS*: *cTBS*) or increase motor cortex excitability (*intermittent TBS*: *iTBS*) for up to 40 min following rTMS as shown in a significant reduction or enhancement of MEP amplitudes (Huang et al., 2005). The maximal change in cortical excitability has been shown to occur approximately 10 min after TBS (Huang et al., 2005). The rest period between TBS intervention and beginning of the “post-TBS” block was, therefore, chosen as 10 min.

## 3 Results

**Fig. 2:**
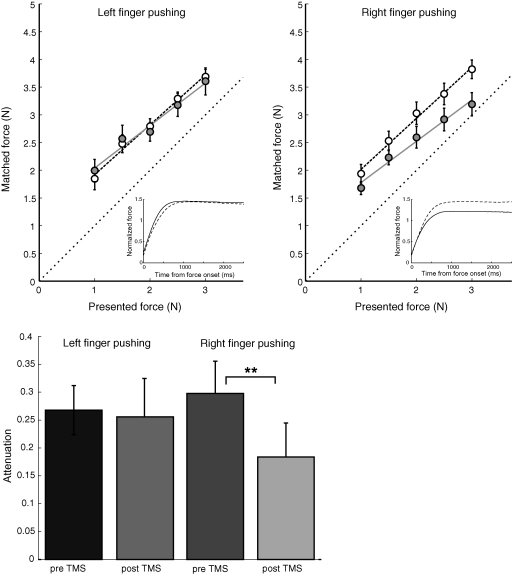
Effects of cTBS on attenuation. Matching force generated using the left index finger (left upper panel) or the right index finger (right upper panel) before (open circles, dotted line) and after (filled circles, solid line) cTBS as a function of the externally generated target force. Error bars indicate S.E. across participants. Dotted line represents perfect performance. Insets show averaged force traces for the mean matching force, aligned to force onset and normalized by target force. The dotted line shows force traces pre-TBS, solid lines represent force profiles after the cTBS intervention. Lower panel: bars representing the amount of attenuation (attenuation index, see Section 2) before and after cTBS for the left and the right finger generating the matching force. **p < 0.05, two-tailed paired *t*-test.

In the pre-TBS blocks, all participants consistently applied a greater force when matching the externally applied target force, that is they consistently underestimated the force they were applying to themselves (Fig. 1C shows a sample trace, Fig. 2 shows group data as well as averaged and normalised force profiles). Significant attenuation was observed pre-TBS in both groups when the matching force was generated with either the left (cTBS 26.8% attenuation, iTBS 28.2%, *p* < 0.002 both groups, two tailed *t*-test) or the right index finger (cTBS 29.9%, iTBS 28.2%, *p* < 0.001 both groups, two tailed *t*-test)

### 3.1 Effects of continuous TBS on attenuation

For the group that received continuous TBS, a repeated measures two-way (cTBS: pre versus post-TBS, active finger: left versus right) ANOVA of attenuation showed a significant interaction (*F*(1,7) = 6.62, *p* = 0.037) (Fig. 2). This interaction arose from the subjects having significantly less attenuation after they received TBS when the matching force was generated with the right finger but no significant difference between pre- and post-TBS performance when using the left index finger to generate the matching force. Therefore, we saw a reduced attenuation and improved performance when the finger contralateral to the cTBS (right index) was required to actively generate the force. No change in attenuation was seen when the contralateral finger was passive and the ipsilateral finger generated the force (left index).

Extending the ANOVA to include target force as a factor resulted in a non-significant three-way interaction term (cTBS × active finger × target force: *F*(4,28) = 0.28, *p* = 0.89), indicating that the effect of cTBS on attenuation did not depend on force level.

### 3.2 Effects of intermittent TBS on attenuation

**Fig. 3:**
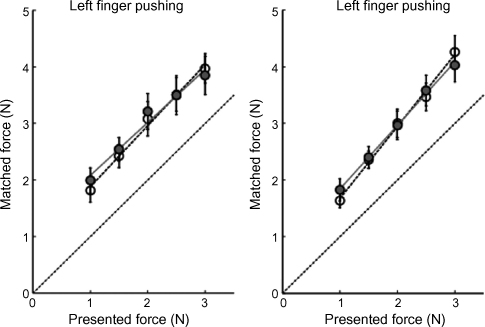
Effects of iTBS on attenuation. Matching force generated using the left index finger (left upper panel) or the right index finger (right upper panel) before (open circles, dotted line) and after (filled circles, solid line) iTBS as a function of the externally generated target force. Error bars indicate S.E. across participants. Dotted line represents perfect performance.

A repeated measures ANOVA showed no effect of either iTBS (*F*(1,7) = 0.8, *p* = 0.4) or active finger (*F*(1,7) = 0.05, *p* = 0.83) and no significant interaction (*F*(1,7) = 0.03, *p* = 0.86). Intermittent TBS, therefore, did not alter the amount of attenuation seen during the task, regardless of which finger was generating the matching force (Fig. 3).

## 4 Discussion

In this study we have replicated the previous finding (Bays et al., 2005, 2006; Shergill et al., 2003) that the sensation of force in a passive digit is attenuated when that force is self-generated. In addition we show that application of *continuous* “Theta-Burst Stimulation” (cTBS) over the primary motor cortex reduces the attenuation of such self-generated forces. In other words, subjects who had received a continuous burst of TBS over motor cortex contralateral to the finger that generated the matching force were more accurate in reproducing the target force. This improved performance after TBS can be interpreted as a lack of normal sensory attenuation due to a diminished sensory predictive process after the TBS intervention.

In the experiment we controlled for non-specific effects of TBS by performing the task with the finger contralateral to the TBS as both the passive finger, which experiences the forces produced by the torque motor or as the active finger, which produces the matching force. Only in the latter condition is a motor command generated by the affected hemisphere. Therefore, the situation in which the finger ipsilateral to the stimulation site matches the target force serves as a control condition and obviates the need for a further sham or control site TMS. The fact that improvement in the task was only observed when the right finger was active excludes the possibility that attenuation occurred because of impaired sensory processing due to current spread to primary sensory areas. Moreover, the results can not be interpreted as a diffuse “motor impairment” or weakness resulting from TBS, as subjects were capable of scaling the matching force as a function of the target force and at high target force levels were capable of producing enough force that attenuation would easily be observable for low target force levels (Fig. 2b).

Many studies have shown that tactile sensation is reduced in a moving body part (Angel & Malenka, 1982; Chapman, Bushnell, Miron, Duncan, & Lund, 1987). For example, detection thresholds for electrical stimuli are raised in a moving finger compared to the finger at rest (Williams, Shenasa, & Chapman, 1998). However, this attenuation reflects a non-specific gating of all sensation in an active body part, whereas in this study we have examined the attenuation of self-generated sensation in a passive body part. This latter attenuation has been shown to be time-locked to the expected time of contact and does not result from movement alone (Bays et al., 2005).

The reduced attenuation in the cTBS group suggests that TBS over M1 induced a mismatch between predicted and actual motor output resulting from changes in cortical excitability resulting in an improved performance at the matching task. This interpretation is consistent with previous findings where the mismatch between predicted and actual sensory consequences was created by introducing spatial or temporal delays between action and effect (Blakemore et al., 1999) or when an action was evoked by TMS (finger twitch) and was thus not predictable (Chronicle & Glover, 2003) and attenuation was strongly diminished. Interestingly, our result resembles the one found in schizophrenic patients in the same task: patients showed a significantly lower amount of attenuation compared to age-matched healthy control subjects. The finding was interpreted as an indication of a dysfunctional sensory predictive mechanism in schizophrenia (Shergill, Samson, Bays, Frith, & Wolpert, 2005).

Although iTBS has been shown to facilitate M1, we did not see any change in attenuation in this condition. However, whereas the effects of cTBS have been shown to be robust across subjects and long lasting iTBS is less robust across subjects and often only lasts a short time after stimulation (Huang et al., 2005).

The exact mechanism by which the cTBS intervention caused a weakening of the predictive process cannot be fully determined from our data. TBS must have a differential effect on the populations of neurones that generate motor output than on those pathways that are involved in generating an efference copy of the motor command.

One possibility is that efference copy signals do not arise as a direct copy of the output of M1 but is generated in areas upstream of M1 before the motor command reaches M1. The change in the excitability of M1 due to TBS then produces a mismatch between output force and efference copy. This assumption, however has to remain speculative since we cannot exclude the possibility that the observed effects of cTBS are due to remote effects resulting from the cortical stimulation over the primary motor cortex. Recent studies show that rTMS over M1 can activate a large cortical network including basal ganglia, premotor and cerebellar regions that are strongly connected with M1 (Bestmann, Baudewig, Siebner, Rothwell, & Frahm, 2004). These remote effects could as well be the cause of weakening of the predictive mechanisms, for example by causing a “virtual lesion” in the cerebellum. Theoretical considerations, imaging data and lesion studies suggest that the cerebellum is functionally best suited to implement internal models which are in turn crucial for predicting the sensory consequences of one's own actions (Blakemore, Frith, & Wolpert, 2001; Kawato et al., 2003).

Alternatively, the weakened predictive process due to TBS may be a result of changes in the intrinsic processing of the efference copy signals in the stimulated M1. It has been shown previously that TBS affects the excitability of intracortical interneurons (Huang et al., 2005), which may lead to an attenuated influence of the efference copy on the corticospinal motor output and thus, reduce sensory attenuation.

In summary we show an improvement in the perception of self-generated tactile stimuli following continuous Theta-Burst Stimulation of primary motor cortex. The effect was only observed when the action was performed with finger contralateral to the stimulated motor cortex and only when an inhibitory TBS protocol was used. We conclude that TBS over M1 has interfered with the sensory attenuation mechanism by creating a mismatch between the predicted and the actual sensory feedback arising from an action.

While the exact site of action remains unknown, we can provide evidence that TBS has a differential effect on the populations of neurones that generate motor output than on those pathways that are involved in generating an efference copy of the motor command. Moreover, we demonstrate that cTBS is a powerful tool to create “virtual” lesions in M1 to examine performance in behavioural tasks.
